# Mitochondrial genome analysis of *Ectophasia roundiventris* (Diptera, Tachinidae)

**DOI:** 10.1080/23802359.2017.1357447

**Published:** 2017-07-26

**Authors:** Xin Li, Shuangmei Ding, Peng Hou, Xiaoyan Liu, Chuntian Zhang, Ding Yang

**Affiliations:** a College of Life Science, Shenyang Normal University, Shenyang, China;; b College of Plant Protection, China Agricultural University, Beijing, China;; c College of Plant Science and Technology, Huazhong Agricultural University, Wuhan, China

**Keywords:** Tachinidae, phylogeny, mitogenome, Calyptratae, Ectophasia, *roundiventris*

## Abstract

The mitochondrial genome of *Ectophasia roundiventris* (Loew, 1858), the first representative of subfamily Phasiinae, was sequenced and annotated. So far, there are four Tachinidae mitochondrial genomes, here, all of them are used in Neighbour-Join and Maximum Likelihood analyses. The nucleotide composition of *Ectophasia roundiventris* mitochondrial genome was 40.4% of A, 39.0% of T, 11.8% of C, 8.8% of G, 79.4% of A + T content. The codon ATG was the most popular start codon. The conservative stop codon was TAA, COX2, and ND5 terminated with an incomplete stop codon T, while the gene ND4 was ended with stop codon TA.

Tachinid flies are one of the highly diversified families of flies with over 8500 known species distributed worldwide and play an important role in control of pests and balance of ecosystem as enemy of pests (O’Hara et al. 2009; O'Hara 2016; O'Hara and Cerretti 2016). Due to its various morphological features, entomologists face great challenges to identify tachinid flies and pay more attention to the phylogenetic relationships in Tachinidae (Meier et al. 2006; O'Hara 2013; Zhao et al. 2013). Four Tachinidae genomes are available in GenBank database, though there are a great number of partial sequences. Hence, we sequenced mitochondrial genome of *Ectophasia roundiventris* (Loew, 1858), the first representative of subfamily Phasiinae for further research.

Specimens of *Ectophasia roundiventris* were collected in Tang river, Liaoyang City, Liaoning Province, China by Qiang Wang, and identified by Prof. Chuntian Zhang. Specimens are deposited in the Entomological Museum of China Agricultural University, Beijing.

The genomic DNA was extracted from adult’s muscle tissues of the thorax using the DNeasy DNA Extraction kit (TIANGEN, Beijing, China), and sequenced under the next generation sequence technology.

The mitochondrial genome of *Ectophasia roundiventris* contains 22 transfer RNA genes, 13 protein-coding genes (PCGs), two ribosomal RNA genes and non-coding control regions, which were similar with related reports before (Kang et al. 2014; Li et al. 2016; Wang et al. 2016a, 2016b, 2016c).

The nucleotide composition of *Ectophasia roundiventris* mitochondrial genome was 40.4% of A, 39.0% of T, 11.8% of C, 8.8% of G, 79.4% of A + T content. The codon ATG was the most popular start codon shared with ATP6, COX2, COX3, CYTB, ND4, ND4L, and start codon ATT was shared with ND2, ND3, ND5, ND6. Particularly, the ATP8 begins with codon ATC, the COX1 begins with codon TCG, and the ND1 begins with codon TTG. The conservative stop codon TAA was shared with ATP6, ATP8, COX1, COX3, ND2, ND4L, ND6, the stop codon TAG was shared with CYTB, ND1, ND3, and the other two genes, COX2 and ND5, were terminated with an incomplete stop codon T, while the gene ND4 was ended with stop codon TA.

Based on 13 PCGs among seven species including *Lucilia sericata* from Calliphoridae and *Sarcophaga crassipalpis* from Sarcophagidae as outgroups, we conducted a phylogenetic analysis with the Neighbour-Join method (NJ) implemented in Mega 7 (Kumar et al. 2016) and Maximum Likelihood (ML) method in RaxML 7.0.3 (Stamatakis 2006). As NJ tree and ML tree have the complete same topology, only one tree labelled by two kinds of support values was given (Figure 1). In NJ and ML analyses, the outgroups *Lucilia sericata* and *Sarcophaga crassipalpis* form a clade diverged from Tachinidae clades. Subfamily Phasiinae is a basal clade to (subfamily Dexiinae + subfamily Exoristinae). The monophyly of family Tachinidae and subfamily Exoristinae was well supported by NJ and ML trees.

**Figure 1.**
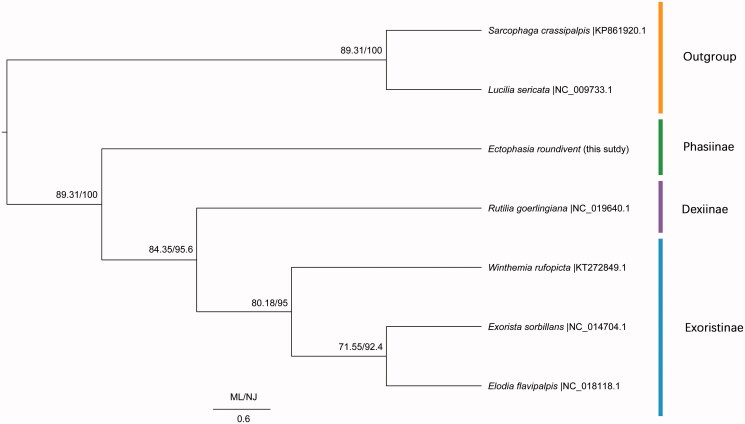
Phylogenetic trees among seven species which consist of five Tachinidae species and two outgroups including Calliphoridae and Sarcophagidae.
